# Enucleation of the Talus

**DOI:** 10.11604/pamj.2016.25.92.9329

**Published:** 2016-10-18

**Authors:** Adil El Alaoui, Nabil Kassou

**Affiliations:** 1 Service de Chirurgie Orthopédique A, Centre Hospitalier Universitaire Hassan II, Fès, Maroc

**Keywords:** Astragalus, enucleation, pinning

## Image in medicine

Enucleation or triple dislocation of the talus is a rare injury; in literature, it represents 2 to 10% of the talar trauma. The prognosis for this type of injury is dominated by the risk of osteonecrosis. We report a case of closed enucleation of the talus, treated in a conservative manner with satisfactory functional outcome. It's a patient of 32 years, victim of a road traffic accident that resulted in an injury of the right foot with pain and total functional impotence. The clinical examination showed a deformation of the foot in valgus, with a protruding talus in the anterolateral leads. The radiography of the ankle objectified anterolateral complete enucleation of the talus with a fracture of the non-displaced lateral malleolus (A). The reduction of the dislocated talus was made by external maneuver, and digital pressure after having set foot in supination and forced equine. The rear foot stability was maintained by a trans-calcaneofibular -talo-tibial pin and two Astragalus-tibial pin cross (B,C). The immobilization of the ankle and the foot was made by a cast boot for two months followed by Rehabilitation. The support has been authorized in the fourth month and the resumption of work in the seventh month after the accident. At 18 months of follow-up, the ankle was painless, stable with a satisfactory mobility. The standard X-rays of the ankle showed no abnormal bone or joint, or sign of necrosis (D, E).

**Figure 1:**
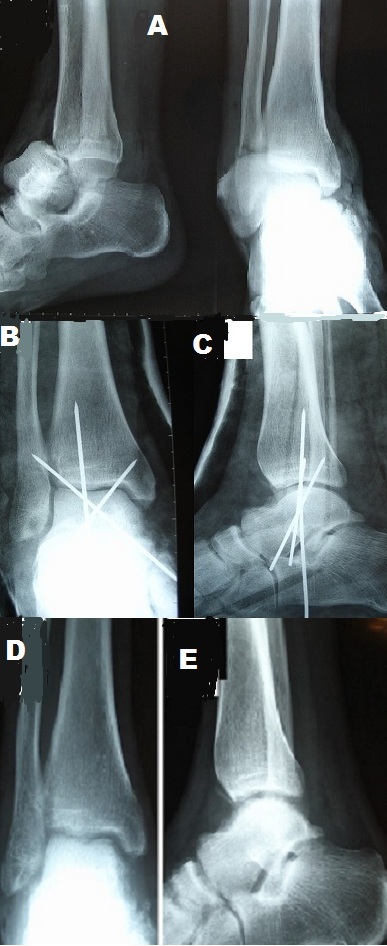
A): the radiography of the ankle objectified anterolateral complete enucleation of the talus with a fracture of the non-displaced lateral malleolus; B, C): the radiography of the ankle objectified trans-calcaneofibular -talo-tibial pin and two Astragalus-tibial pin cross; D,E): the standard clichés of the ankle showed no abnormal bone or joint, or sign of necrosis

